# Does HIV Cause Cardiovascular Disease?

**DOI:** 10.1371/journal.pmed.0030496

**Published:** 2006-11-28

**Authors:** Andrew Carr, Daniel Ory

## Abstract

The authors discuss a study in PLoS Biology that investigated whether the lower levels of HDL cholesterol in patients with HIV may be due to impaired cholesterol efflux from macrophages.

## HIV, Antiretroviral Therapy, and Cardiovascular Disease

Cardiovascular disease is an increasing cause of morbidity in HIV-infected adults receiving antiretroviral therapy (ART). ART, particularly protease inhibitors and to a lesser extent nucleoside analogue reverse transcriptase inhibitors, can adversely affect lipid and glucose metabolism [1]. Moreover, there is a strong correlation between ART duration and the risk of myocardial infarction, an association in part linked to higher plasma levels of total cholesterol and triglyceride and to lower levels of high-density lipoprotein (HDL) cholesterol [2]. Paradoxically, interruption of ART also appears to be associated with an increased short-term risk of cardiovascular disease [3]. These findings suggest that HIV itself may also increase cardiovascular risk, and that control of HIV replication might reduce this risk.

HDL cholesterol levels are reduced in untreated HIV infection [4] and in healthy volunteers exposed for a short term to the HIV protease inhibitors ritonavir-boosted atazanavir and lopinavir [5,6]. In HIV-infected individuals who start ART that effectively suppresses HIV replication, HDL cholesterol levels increase, regardless of whether a protease inhibitor is used [7–9], implying that the HIV effect on HDL cholesterol levels is greater than the ART effect.

## How Does HIV Lower HDL Cholesterol Levels?

The pathogenesis of low HDL cholesterol levels in untreated HIV infection is unknown. In a study recently published in *PLoS Biology*, Mujawar et al. investigated whether these lower levels might be due to impaired cholesterol efflux from macrophages [10], a process mediated by the ABCA1 cell-surface cholesterol transporter. ABCA1 lipidates apoA-I, the major apolipoprotein in HDL, and thus plays a central role in formation of nascent HDL. Mutations in *ABCA1* cause Tangier disease, which is associated with low HDL cholesterol and accelerated atherosclerosis [11].

The researchers found that expression of HIV nef, a protein that enhances HIV replication and infectivity, specifically inhibited ABCA1-dependent cholesterol efflux from macrophages (the precursors of foam cells in atherosclerotic plaque) and re-localized ABCA1 to an exclusive plasma membrane distribution. While *nef*-transfected cells exhibited increased apoA-I binding, apoA-I internalization was blocked, suggesting that nef at the plasma membrane may prevent ABCA1 internalization and subsequent apoA-I lipidation. As cholesterol is required for HIV replication [12], Mujawar et al. next examined whether enhancing cholesterol efflux from HIV-infected macrophages affected HIV replication. Using a liver X receptor (LXR) agonist to transcriptionally upregulate ABCA1, cholesterol efflux was augmented, significantly reducing virion-associated cholesterol and infectivity.

There is a strong correlation between ART duration and the risk of myocardial infarction.

## What Do the Findings Mean?

Mujawar et al. propose that HIV nef redirects cholesterol from an ABCA1-mediated efflux pathway to virus-controlled cholesterol transport in order to ensure sufficient cholesterol for virion assembly. The interruption of a host cholesterol trafficking pathway by an intracellular pathogen is also employed by Toxoplasma gondii, in which endocytosed low-density lipoprotein cholesterol is diverted to a specialized vacuole to support parasite growth and replication [13]. Given that impaired ABCA1 function results in decreased HDL cholesterol and accelerated atherosclerosis [14], the findings of Mujawar et al. provide a possible mechanism to explain low HDL cholesterol in HIV infection and increased cardiovascular risk in HIV-infected adults.

It remains to be determined, however, if this mechanism is the greatest contributor to these low levels. If so, one would expect that HDL cholesterol levels would fall rapidly after primary HIV infection and return to pre-infection levels with effective ART that did not affect lipid metabolism. In addition, the observed reductions might be inversely proportionate to plasma HIV RNA levels. Alternatively, low HDL cholesterol levels may also be caused by ART-induced lipodystrophy. This ART side effect is characterized by peripheral lipoatrophy and relative central adiposity and is strongly associated with low HDL cholesterol levels as well as insulin resistance and hypertriglyceridemia, all features of congenital lipodystrophies in which abnormalities of ABCA1 have not been identified [2,15,16].

## The Strengths and Limitations of the Study

The findings of Mujawar et al. raise several important questions. Is the apparent nef-stimulated decrease in ABCA1 protein levels due to ABCA1 degradation, or possibly due to altered partitioning of ABCA1 into detergent-resistant lipid domains? Does nef physically interact with ABCA1? Use of the myristoylation-deficient nef in this study only suggests that nef must be targeted to the plasma membrane—likely to cholesterol rafts [17]. Furthermore, experiments involving immunoprecipitation of raft-associated proteins must be interpreted with caution, and should be followed up with more rigorous studies (e.g., identification of interaction domains and fluorescence resonance energy transfer) that provide support for a specific ABCA1–nef interaction at the plasma membrane. Finally, might LXR ligands activate target genes, other than *ABCA1*, that could attenuate virion-associated cholesterol? Treatment of HIV-infected ABCA1-deficient macrophages with LXR ligands should establish a definitive role for ABCA1 in countering HIV replication.

## Where To From Here?

The present data provide a clue as to why HIV infection, as well as ART, might accelerate atherosclerosis. The data support the shift away from a paradigm of delaying or stopping ART to reduce the risk of cardiovascular disease and raise the possibility that antiretroviral drugs without direct metabolic effects may actually reduce cardiovascular risk. The data also emphasize the need for clinicians to consider all the metabolic effects (in particular the ratio of total cholesterol to HDL) of each antiretroviral drug, not just total cholesterol levels, in the management of cardiovascular risk in HIV-infected adults.

The study by Mujawar et al. also has important implications for developing new approaches for suppression of HIV replication. Selective LXR modulators under active development for treatment of atherosclerosis may prove useful as ART.

The long-term effects of each antiretroviral drug on HDL cholesterol, as well as other lipid and glycemic parameters, need to be more completely analyzed in randomized trials, as well as in shorter healthy volunteer studies. It remains to be determined whether new antiretroviral drug classes, such as HIV fusion, CCR5, and integrase inhibitors, will be free of these metabolic complications.

## Abbreviations

ART: antiretroviral therapy
HDL: high-density lipoprotein
LXR: liver X receptor
